# Cardiotoxicity risk factors with immune checkpoint inhibitors

**DOI:** 10.1186/s40959-022-00130-5

**Published:** 2022-03-11

**Authors:** Zachary L. Brumberger, Mary E. Branch, Max W Klein, Austin Seals, Michael D. Shapiro, Sujethra Vasu

**Affiliations:** 1 Department of Internal Medicine, Wake Forest University School of Medicine, 1 Medical Center Boulevard, Winston-Salem, NC 27157 USA; 2 Department of Internal Medicine, Section On Cardiovascular Medicine, Wake Forest University School of Medicine, 1 Medical Center Boulevard, Winston-Salem, NC 27157 USA

**Keywords:** Immunotherapy, Cardiotoxicity, Immune checkpoint inhibitors, Programmed death ligand, Atrial fibrillation, Heart failure, Myocarditis, Pericarditis

## Abstract

**Background:**

Checkpoint-inhibitor immunotherapies have had a profound effect in the treatment of cancer by inhibiting down-regulation of T-cell response to malignancy. The cardiotoxic potential of these agents was first described in murine models and, more recently, in numerous clinical case reports of pericarditis, myocarditis, pericardial effusion, cardiomyopathy, and new arrhythmias. The objective of our study was to determine the frequency of and associated risk factors for cardiotoxic events in patients treated with immune checkpoint inhibitors.

**Methods:**

Medical records of patients who underwent immunotherapy with durvalumab, ipilimumab, nivolumab, and pembrolizumab at Wake Forest Baptist Health were reviewed. We collected retrospective data regarding sex, cancer type, age, and cardiovascular disease risk factors and medications. We aimed to identify new diagnoses of heart failure, atrial fibrillation, ventricular fibrillation/tachycardia, myocarditis, and pericarditis after therapy onset. To assess the relationship between CVD risk factors and the number of cardiac events, a multivariate model was applied using generalized linear regression. Incidence rate ratios were calculated for every covariate along with the adjusted *P*-value. We applied a multivariate model using logistic regression to assess the relationship between CVD risk factors and mortality. Odds ratios were calculated for every covariate along with the adjusted *P*-value. Adjusted *P*-values were calculated using multivariable regression adjusting for other covariates.

**Results:**

Review of 538 medical records revealed the following events: 3 ventricular fibrillation/tachycardia, 12 pericarditis, 11 atrial fibrillation with rapid ventricular rate, 0 myocarditis, 8 heart failure. Significant risk factors included female gender, African American race, and tobacco use with IRR 3.34 (95% CI 1.421, 7.849; *P* = 0.006), IRR 3.39 (95% CI 1.141, 10.055; *P* = 0.028), and IRR 4.21 (95% CI 1.289, 13.763; *P* = 0.017) respectively.

**Conclusions:**

Our study revealed 34 significant events, most frequent being pericarditis (2.2%) and atrial fibrillation (2.0%) with strongest risk factors being female gender, African American race, and tobacco use. Patients who meet this demographic, particularly those with planned pembrolizumab treatment, may benefit from early referral to a cardio-oncologist. Further investigation is warranted on the relationship between CTLA-4 and PD-L1 expression and cardiac adverse events with ICIs, particularly for these subpopulations.

## Background

Checkpoint-inhibitor immunotherapies have had a profound effect in the treatment of numerous malignancies. With the advent of humanized anti-cytotoxic T lymphocyte antigen 4 (CTLA-4) antibody, melanoma patients have seen a significant increase in 5-year survival from approximately 10% to 20–26% [1]. In the KEYNOTE-010 Trial, pembrolizumab (an antibody against PD-L1) was shown to increase median overall survival to 12.7 months as compared to 8.5 months with docetaxel [2]. The list of malignancies that can be treated with these antibodies continues to grow and includes bladder cancer, melanoma, lung cancer, renal cell cancer, head and neck cancers, hepatocellular carcinoma, and more [3]. Currently, there are two major pathways that these new agents target. The first involves CTLA-4, a protein expressed by T-cells which when bound to B7-1 and B7-2 on antigen-presenting cells, negatively regulates T-cell function thereby evading eradication [4]. The second involves PD1, a transmembrane receptor expressed on activated lymphocytes that binds to other transmembrane proteins’ programed cell death protein 1 and 2 (PD-L1 and PD-L2). When PD-L1 binds to the PD1 of a T-cell, it leads to T-cell inhibition. Both CTLA-4 and PD-L1 have been found to be expressed on many different neoplastic cells and this is thought to be a major mechanism of immune evasion [2, 5]. The agents durvalumab, nivolumab and pembrolizumab target PD-L1, while ipilimumab targets the CTLA-4 pathway.

Common side effects of these medications include: colitis, dermatitis, endocrinopathies, hepatitis, and pneumonitis [6, 7]. Because cardiac health monitoring is not routinely done with these patients, it is possible that adverse cardiac events are underreported. Since the early 2000s, cardiotoxic effects have been discovered in murine models. Initial studies found that PD-1 deficient mice developed dilated cardiomyopathy [8, 9]. Later studies found that PD-1 deficient mice also began to suffer from severe myocarditis [10, 11]. Indeed, myocarditis has been reported in patients treated with these immunotherapy agents. According to 2016 pharmacovigilance analysis, the incidence of myocarditis was 0.09% in those who received nivolumab. In patients who received a combination of ipilimumab and nivolumab, there was about a 0.27% incidence [7, 12, 13]. There have been case reports of other cardiotoxic effects including pericarditis, pericardial effusion, cardiomyopathy, and new-onset arrhythmias [14–17]. Given the growing concern for these toxicities, we sought to determine the frequency of and risk factors for cardiotoxic events in patients treated with immune checkpoint inhibitors.

## Methods

### Study design

We utilized the local EPIC database at Wake Forest Baptist Clinical Cancer Center (an NCI-designated cancer center). Medical record numbers were pulled of those patients who had received immunotherapies including: pembrolizumab, ipilimumab, durvalumab, or nivolumab. The earliest record of chemotherapy started on October 11^th^, 2012 and chart review data was collected through the month of October 2019. Each patient chart was reviewed for: start date of each chemotherapy, International Classification of Disease (ICD) 9/10 codes for coexisting conditions (hypertension, diabetes mellitus, symptomatic heart failure), concurrent prescription medications (ACEIs, ARBs, beta-blockers, calcium channel blockers, diuretics, and statins), and hospitalizations while on immunotherapy that included new or worsening diagnoses of heart failure, atrial fibrillation with rapid ventricular response, ventricular fibrillation/tachycardia, myocarditis, or pericarditis. In further detail, heart failure was defined by new ICD 9/10 code, evidence of impaired filling on echocardiography with symptoms of volume overload: new/worsening edema, dyspnea, orthopnea, shortness of breath with radiographic evidence of interstitial edema, or worsening EF in those with pre-existing heart failure. Assessment of ejection fraction was completed by licensed sonographers for general clinical practice.

### Covariates

Patient demographics and cardiovascular disease (CVD) risk factors were obtained from the EMR as covariates which included: age, gender, race, body mass index (kg/m^2^), hypertension, diabetes mellitus type II and smoking status. Presence of hypertension and diabetes mellitus type II was derived from ICD 9/10 codes. The diagnostic criteria for hypertension evolved multiple times during the study period, but was generally understood to be an elevated blood pressure at the time of diagnosis, taken on two readings on two separate occasions. The diagnostic criteria for diabetes mellitus type II was hemoglobin A1c greater than 6.5%, fasting glucose greater than 125 mg/dl, or random plasma glucose greater than 200 mg/dl. Smoking status was defined as either non-smoker or any history of tobacco use, former or current.

### Outcomes

The primary outcome in this study was a cardiac event after immune checkpoint inhibitor treatment which was a composite of the following incident diagnoses: myocarditis, pericarditis, atrial fibrillation with rapid ventricular response, ventricular fibrillation/tachycardia, heart failure with preserved ejection fraction, and heart failure with reduced ejection fraction.

### Statistical methods

In this study, we compared demographic and CVD risk factor prevalence in patients who developed cardiac events after exposure to immune checkpoint inhibitors. Continuous variables were compared using t-test. Categorical variables were compared using chi-square analysis. To assess the relationship between CVD risk factors and the number of cardiac events, a multivariate model was applied using generalized linear regression, with the model fit to a negative binomial distribution to address potential over dispersion of the count data. Incidence rate ratios were calculated for every covariate along with the adjusted *P*-value. We applied a multivariate model using logistic regression to assess the relationship between CVD risk factors and mortality. Odds ratios were calculated for every covariate along with the adjusted *P*-value. Adjusted *f*values were calculated using multivariable regression adjusting for other covariates shown in the results. Analyses were performed with RStudio version 1.1453 (© 2009–2018 RStudio, Inc.) and SAS 9.4 (The SAS Institute, Cary, North Carolina). Significance testing was two-sided, with *P* < 0.05 considered significant.

## Results

Demographic data of those with and without cardiac events can be found in Table 1. There was a significantly higher percentage of women experiencing cardiac events compared to men (8.1% vs. 2.9%; *P* = 0.011) as well as a higher percentage of African Americans with cardiac events than Caucasians with cardiac events (12% vs. 4%; *P* = 0.02). Patients undergoing treatment with Pembrolizumab (*n* = 243) had higher cardiac events rates compared to Nivolumab (*n* = 220) (7% vs. 4%).

**Table 1:** Demographics of Patients with and without Adverse Cardiac Events with Immunotherapy

	Persons with Cardiac Events *n* = 26	Persons without Cardiac Events *n* = 512	*P* Value
**Demographics**			
Age, years [mean** ± **SD]	65 ± 8	66 ± 11	0.881
Gender (F) n (%^1^)	16 (8.1%)	180 (92%)	0.011^a^
Race			
Caucasian n (%^1^)	19 (4%)	449 (96%)	0.017^b^
African American n (%^1^)	7 (12%)	50 (88%)	-
Other	0	13 (2.5%)	-
**Cardiovascular Disease Risk Factors**			
BMI (kg/m^2^)	25 ± 6	26 ± 7	0.308
Hypertension n (%^1^)	14 (4%)	307 (96%)	0.678
Diabetes Mellitus, Type II n (%^1^)	2 (7.7%)	87 (17%)	0.339
Smoker n (%^1^)	22 (6%)	332 (94%)	0.062
**Immune Checkpoint Inhibitors**			
Pembrolizumab n (%^1^)	17 (7%)	226 (93%)	0.055
Nivolumab n (%^1^)	8 (4%)	212 (96%)	0.383
Ipilimumab n (%^1^)	0	39 (100%)	-
Durvalumab n (%^1^)	1 (3%)	35 (97%)	0.847
**Cardiovascular Medications prior to starting ICI**			
Beta Blockers n (%^1^)	11 (7.2%)	141 (92.8%)	0.103
ACE/ARB n (%^1^)	5 (4.1%)	118 (95.9%)	0.651
Calcium Channel Blocker n (%^1^)	6 (5.4%)	106 (94.6%)	0.771
Diuretic n (%^1^)	6 (6.9%)	81 (93.1%)	0.327
Statin n (%^1^)	10 (5.9%)	159 (94.1%)	0.427
No Cardiovascular Medications	6		

There were 26 patients with cardiac events and 34 events total

^1^Represents percentage of subgroup population with or without occurrence of event

^a^Chi-square comparing Females and Males

^b^Chi-square comparing Caucasian and African American race only

After review of 538 medical records, we identified 34 cardiac events after exposure to immune checkpoint inhibitors with a mortality rate of 53% (Table 2). The most commonly reported events were pericarditis (2.2%), atrial fibrillation with rapid ventricular response (2.0%) and heart failure (1.5%). Of the 39 patients with ipilimumab, no adverse cardiac events were found. The percentage of adverse cardiac events in those treated with pembrolizumab was substantially higher than those treated with nivolumab or durvalumab. In total, 21 events occurred in total out of the 243 (8.6%) patients treated with pembrolizumab, with pericarditis (2.9%) and atrial fibrillation with rapid ventricular response (2.5%) being most common. A total of 11 adverse events where found in the 220 patients (5%) treated with nivolumab; roughly half the event rate as compared to pembrolizumab. In those 36 patients treated with durvalumab, only two adverse events were found. In analyzing the differences in event rates between malignancy types, the majority of adverse cardiac events occurred in those with non-small cell lung cancer with a total of 29 total events amongst the 254 (11.4%) patients. By comparison, only 2 adverse events occurred in the group of 30 patients with small cell lung cancer (6.7%). Only a couple of events were found in those with all other malignancy types (endometrial, esophageal, leukemia, lymphoma, melanoma, mesothelioma, multiple myeloma, neuroendocrine, ovarian, and parotid). Heart failure was found in 2.4% of those treated with pembrolizumab as compared to only 0.5% of those in the nivolumab group. There were 45 patient records with both pre-treatment and treatment echocardiogram data showing an average decrease in left ventricular ejection fraction of 1.9%.

**Table 2:** Incidence of adverse cardiac events with immunotherapy

	Atrial Fibrillation with RVR	HFpEF	HFrEF	Myocarditis	Pericarditis	VF/VT	Total Cardiac Events (*N* = 34) *N* (% rate per agent)
Immune Checkpoint Inhibitor Agent							
*Durvalumab* (*n* = 36) *N* (%)^1^	1 (2.8%)^1^	0	1 (2.8%)^1^	0	0	0	2 (5.6%)
*Ipilimumab* (*n* = 39) *N* (%)^1^	0	0	0	0	0	0	0
*Nivolumab* (*n* = 220) *N* (%)^1^	4 (1.8%)^1^	0	1 (0.5%)^1^	0	5 (2.3%)^1^	1 (0.5%)^1^	11 (5%)
*Pembrolizumab* (*n* = 243) N (%)^1^	6 (2.5%)^1^	2 (0.8%)^1^	4 (1.6%)^1^	0	7 (2.9%)^1^	2 (0.8%)^1^	21 (8.6%)
Type of Malignancy^a^							*N* (% rate per malignancy subtype)
*Endometrial* (*n* = 2) *N* (%)^1^	0	0	1 (50%)^1^	0	0	0	1 (50%)
*Esophageal* (*n* = 4) *N* (%)^1^	1 (25%)^1^	0	0	0	0	0	1 (25%)
*Non-Small Cell Lung* (*n* = 254) *N* (%)^1^	10 (3.9%)^1^	2 (0.8%)^1^	3 (1.2%)^1^	0	12 (4.7%)^1^	2 (0.8%)^1^	29 (11.4%)
*Small Cell Lung* (*n* = 30) *N* (%)^1^	0	0	1 (3.3%)^1^	0	0	0	1 (3.3%)
*Squamous Cell Carcinoma* (*n* = 2) *N* (%)^1^	0	0	1 (50%)^1^	0	0	1 (50%)^1^	2 (100%)
*Total* (*n* = 538) *N* (%)^2^	11 (2.0%)^2^	2 (0.4%)^2^	6 (1.1%)^2^	0 (0%)^2^	12 (2.2%)^2^	3 (0.6%)^2^	

*RVR* rapid ventricular response, *HFpEF/HFrEF* heart failure with preserved ejection fraction/ reduced ejection fraction, *VF/VT* ventricular fibrillation/ tachycardia

^a^Other malignancies found in analysis without incidence of events included: leukemia, lymphoma, melanoma, mesothelioma, multiple myeloma, neuroendocrine, ovarian, and parotid

^1^% rate per subgroup population

^2^% rate per study population

**Fig. 1:**
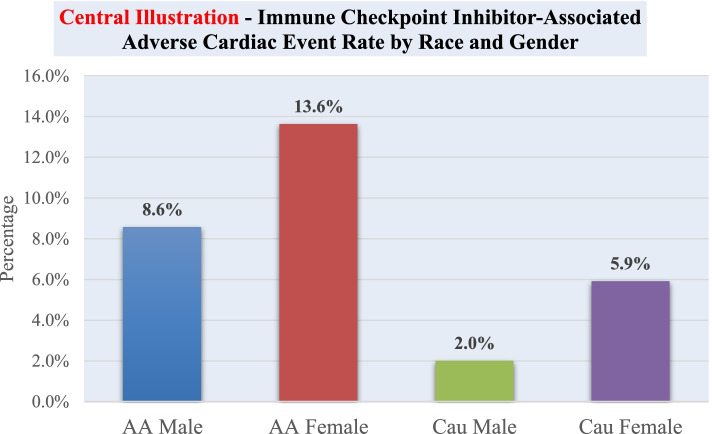
Percentage of adverse cardiac event (atrial fibrillation with rapid ventricular response, heart failure with preserved/reduced ejection fraction, myocarditis, pericarditis, ventricular fibrillation/tachycardia) out of respective study population treated with immune checkpoint inhibitors. Total population for each category: African American (AA) Male 35, AA Female 22, Caucasian (Cau) Male 299, Cau Female 169. Fisher’s Exact *P* = 0.057 between AA and Cau male and *P* = 0.176 between AA and Cau females

After assessment of each CVD risk factor and the association of composite cardiac events, female gender was found to have significant increased incidence rate ratio of developing the composite cardiac event outcome in a multivariate model (IRR 3.340; 95% CI 1.421, 7.849; *P* = 0.006) (Table 3). However, there was no significant increased risk in mortality (OR 1.108; 95% CI 0.769, 1.595) (Table 4). African American race was also found to be significantly associated with the primary outcome in a multivariate model [IRR 3.388 (Caucasian reference); 95% CI 1.141, 10.055; *P* = 0.028] (Table 3). Again, we found no significant increased risk in mortality (OR 1.149; 95% CI 0.647, 2.04) (Table 4). A visual comparison between race and gender adverse cardiac event rate can be found in the Fig. 1. Additionally, smoking was found to be significantly associated with the primary outcome in a multivariate model (IRR 4.21; 95% CI 1.289, 13.763; *P* = 0.017) (Table 3). There was no significant increased risk in mortality with regards to smoking (OR 1.285; 95% CI 0.883, 1.871) (Table 4). We found no statistically significant increased odds of developing cardiac events with age, BMI, hypertension, or diabetes mellitus type II. Each one point increase in BMI was significantly associated with decreased odds of mortality (OR 0.937; 95% CI 0.910, 0.965; *P* = < 0.0001) (Table 4).

**Table 3:** Risk of cardiac events associated with CVD risk factors

Covariate	Incidence Rate Ratio (95%CI)	Adjusted *P* Value^a^
*Age*	1.012 [0.970,1.056]	-
*Gender*	3.340 [1.421, 7.849]	0.0057
*Race*^b^ *(Caucasian reference)*	3.388 [1.141, 10.055]	0.0279
*BMI*	1.026 [ 0.957, 1.010]	-
*Hypertension*	1.029 [0.402, 2.637]	-
*Diabetes Mellitus Type II*	0.327 [0.075, 1.435]	-
*Smoker*	4.212 [1.289, 13.763]	0.0173

^a^Adjusted for age, race and gender

^b^Only assessed African Americans and Caucasians

**Table 4:** Risk of mortality associated with CVD risk factors

Covariate	Odds Ratio (95%CI)	Adjusted *P* Value^a^
*Age*	0.997 (0.981,1.015)	-
*Gender* *(Female reference)*	1.108 [0.769,1.595]	-
*Race*^b^ *(African American reference)*	1.149 [0.647,2.040]	-
*BMI*	0.937 [0.910,0.965]	< .0001
*Hypertension*	1.244 [0.852,1.818]	-
*Diabetes Mellitus Type II*	1.369 [0.835,2.244]	-
*Smoker*	1.285 [0.883,1.871]	-

^a^Adjusted for age, race and gender

^b^Only assessed African Americans and Caucasians

## Discussion

Our study revealed 34 significant cardiac events amongst all those treated with checkpoint-inhibitor immunotherapy. The most frequently reported events in this study included pericarditis (2.2%), atrial fibrillation (2.0%), and heart failure (1.5%). These infer an incidence rate of 8.5 per 1000 person years for pericarditis, 7.8 per 1000 person years for atrial fibrillation, and 5.7 per 1000 person years for heart failure while being treated with ICIs. Comparatively, general population studies have found a 4.4% incidence rate of heart failure over an 11.5 year period and pericarditis incidence of 27.7 cases per 100,000 persons [18, 19]. The ARIC study demonstrated an incidence rate for atrial fibrillation in Forsyth County (where Wake Forest Baptist is located) of 5.2 per 1000 person years for whites and 2.9 per 1000 person years for African Americans [20]. Thus, it appears our cohort of patients being treated with ICIs had a marked increase in incidence of cardiac adverse events compared to the general population.

We found that of the four immunotherapies, patients who underwent treatment with pembrolizumab had the highest frequency of adverse events making up 21 of the 34 total events. Given the large discrepancy in sample size between pembrolizumab (243) and ipilimumab (39) or durvalumab (36), it would seem premature to compare the event rates between these groups. However, the sample size between nivolumab and pembrolizumab was similar at 243 and 220, respectively. Interestingly, those treated with pembrolizumab had nearly double the event rate as compared to nivolumab. Atrial fibrillation with RVR was seen in 2.5% of those in the pembrolizumab arm as compared to 1.8% of those treated with nivolumab. Pericarditis was reported in 2.9% of those treated with pembrolizumab versus 2.3% with nivolumab. All this would suggest that pembrolizumab may warrant closer monitoring.

In 2019, Salem et al. published a large, multi-center, international, retrospective pharmacovigilance study comparing adverse cardiac events in those treated with ICIs to a control reference population of over 16 million patients [21]. They reported myocarditis in 0.39% of those treated with ICIs compared to 0.03% in the reference population. In addition, further analysis found event rates between the ICI and reference groups for pericardial disease, supra-ventricular arrhythmias, and heart failure to be 0.3% vs 0.08%, 0.71% vs 0.42%, and 0.72% vs 0.87%, respectively. Our event frequency in comparison appears alarmingly higher, excluding myocarditis (no events in our findings). The difference likely lies on how data was collected, wherein Salem et al. relied on reported events, we thoroughly adjudicated patient events in the electronic medical record.

A retrospective study published by Escudier et al. in 2017 looked more closely at patients from two cardio-oncology clinics highly suspected to have ICI-related cardiotoxicity [22]. They reported data from 30 patients, 23 were male (77%) with a mean age of 72, finding left ventricular systolic dysfunction in 79%, Takotsubo syndrome-like appearance in 14%, atrial fibrillation in 30%, ventricular arrhythmia in 27%, conduction abnormalities in 17%, and pericardial effusion in 7%. In our study, we also had more males than females (64% to 36%) even as compared with this study, but our mean age was younger at 65. It should be noted that only 10% of the patients in the Escudier study were treated with pembrolizumab and instead the majority 87% were treated with ipilimumab and/or nivolumab.

In early 2020, Oren et al. looked at 3,326 patients who received ICIs and formulated Cox proportional-hazard models for various risk factors [23]. They found both obesity and hypercholesterolemia to be associated with lower all-cause mortality with respective hazard ratios of 0.65 (95% CI 0.55–0.77, *P* < 0.001) and 0.8 (95% CI 0.72–0.89, *P* < 0.001). Additionally, they reported hypertension and smoking hazard ratios of 1.32 (95% CI 1.17–1.49, *P* < 0.001) and 1.17 (95% CI 1.10–1.76, *P* = 0.006), respectively. In our risk factor analysis, CVD risk factors of age, hypertension, and diabetes mellitus type II were not found to be significantly associated with cardiac events or mortality. Similar to Oren et al., we found obesity to be associated with lower mortality. We also found smoking to be associated with increased cardiac events but without association to mortality. There was, however, some paucity of data with 10% of patients listed as unknown smoking status.

African Americans and women were found to be significantly associated with increased risk of cardiac events after exposure to immune checkpoint inhibitors. This may relate to decreased access to preventative care, follow-up care, or differences in shared variants between African Americans and those of European descent as it relates to PD-L1 expression and/or inflammatory response. African Americans have been described as having elevated inflammatory markers with recent evidence that suggests an association between inflammation and worsened cancer survival [24–26]. Of note, a recent study did not find a difference in the expression of PD-L1 and PD-L2 between lung tumors from African Americans and those of European ancestry, but did find a significant increase in CTLA-4 expression on African American lung tumors [27]. PD-L1 expression has been found to occur with higher frequency in females in studies for both non-small cell lung cancer and oral squamous cell carcinoma [28, 29]. These sex and racial differences in immune response signaling pathways could explain the increase in adverse events found in our study, as the mechanisms for immune-related adverse events during treatment with ICIs are thought to be related to changes in patterns of T and B cell expression [30]. Additionally, these mechanisms have also been linked to autoimmune diseases [30] which occur more frequently in females and African Americans.

We did not find any cardiac events in those treated with ipilimumab. This finding could suggest that cardiotoxic events occur less frequently with this agent, as there were two events that occurred in the 36 patients treated with durvalumab. However, this is likely insignificant and represents small sample size for this specific agent, as multiple reports have reported adverse cardiac events in those treated with ipilimumab [6, 7, 14, 31].

### Study limitations

As with many retrospective studies, intervals between clinical visits/evaluations, interventions (including medications), imaging, and echocardiography could not be standardized. Unfortunately, many patients lacked complete echocardiogram data which could have confirmed more or fewer cases of heart failure, pericardial effusion/pericarditis, or myocarditis. There was a paucity of troponin and advanced cardiac imaging, which may have revealed that more of the reported cases of pericardial effusion/pericarditis events were, in fact, instances of myocarditis. In addition, it is likely that many events have been missed as there were numerous patients who moved out of network or passed away outside of the hospital. Given this lack of data, it is more-than-likely that the rates of these events were even higher than we report. Importantly, there is an additive effect of ICI use in terms of risk of myocarditis and this was not explored [32]. However, this study included a robust sample size and broad evaluation of cardiac events. In addition, this is the first study to find a difference in race and gender in terms of risk for cardiac events at exposure to ICIs.

## Conclusion

In our study we found that patients treated with pembrolizumab have higher incidence of cardiac events. In addition, female gender, African American race, and tobacco use were significantly associated with increased risk of cardiac events after exposure to immune checkpoint inhibitors. Further investigation is warranted on the relationship between CTLA-4 and PD-L1 expression and cardiac adverse events with ICIs, particularly for these subpopulations. Patients who meet this demographic, particularly those with planned pembrolizumab treatment, may benefit from early referral to a cardio-oncologist.

## Data Availability

The datasets used and/or analyzed during the current study are available from the corresponding author on reasonable request. The majority of data and materials is available in the results, tables, and figures.

## Abbreviations

AA: African American
ACEI: Angiotensin-Converting Enzyme Inhibitor
ARB: Angiotensin II Receptor Blockers
BMI: Body Mass Index
Cau: Caucasian
CI: Confidence Interval
CTLA-4: Cytotoxic T Lymphocyte Antigen
CVD: Cardiovascular Disease
EMR: Electronic Medical Record
HFpEF: Heart Failure with Preserved Ejection Fraction
HFrEF: Heart Failure with Reduced Ejection Fraction
ICD: International Classification of Disease
ICI: Immune Checkpoint Inhibitors
NCI: National Cancer Institute
OR: Odds Ratio
PD1: Programmed Cell Death Protein 1
PD-L1: Programmed Death Ligand 1
PD-L2: Programmed Death Ligand 2
RVR: Rapid Ventricular Response
VF: Ventricular Fibrillation
VT: Ventricular Tachycardia
